# Gene Therapy in the Light of Lifestyle Diseases: Budesonide, Acetaminophen and Simvastatin Modulates rAAV Transduction Efficiency

**DOI:** 10.3390/ph17091213

**Published:** 2024-09-14

**Authors:** Żaneta Słyk, Natalia Stachowiak, Maciej Małecki

**Affiliations:** 1 Department of Applied Pharmacy, Faculty of Pharmacy, Medical University of Warsaw, 02-091 Warsaw, Poland; 2 Laboratory of Gene Therapy, Faculty of Pharmacy, Medical University of Warsaw, 02-091 Warsaw, Poland

**Keywords:** rAAV, drugs, gene therapy, acetaminophen, simvastatin, budesonide, pharmacokinetics/pharmacodynamics

## Abstract

Recombinant AAV (rAAV) vectors are increasingly favored for gene therapy due to their useful features of vectorology, such as transfection of dividing and nondividing cells, the presence of tissue-specific serotypes, and biosafety considerations. This study investigates the impact of commonly used therapeutic drugs—acetaminophen, budesonide, and simvastatin—on rAAV transduction efficiency in HEK-293 cells. Cells were transduced with an AAV mosaic vector under the control of a cytomegalovirus (CMV) promoter encoding green fluorescent protein (GFP). Transduction efficiency was assessed by qPCR and fluorescent microscopy. Analysis of functional interactions between genes potentially involved in rAAV transduction in drug-exposed cells was also performed. This study showed a clear effect of drugs on rAAV transmission. Notably, acetaminophen enhanced transduction efficiency by 9-fold, while budesonide and simvastatin showed 2-fold and 3-fold increases, respectively. The gene analysis illustrates the possible involvement of genes related to cell membranes in the potentiation of rAAV transduction induced by the drugs under investigation. Attention should be paid to *S100A8*, which is a common drug-modified gene for drugs showing anti-inflammatory effects (budesonide and simvastatin), demonstrating an interaction with the gene encoding the receptor for AAV (HGFR). This study underscores the significance of assessing rAAV pharmacokinetics/pharmacodynamics (PKs/PDs) and drug–gene therapy interactions in optimizing gene therapy protocols.

## 1. Introduction

Recombinant adeno-associated virus (rAAV) vectors are now among the well-recognized gene therapy solutions in the field of viral methods of gene transfer [1]. There is a substantial body of work planned for experimental and clinical applications of rAAV vectors. The great interest in rAAV vectors is further substantiated by the official registration of rAAV preparations as human drugs. Hemgenix^®^ and Roctavian^®^ are approved by both the U.S. Food and Drug Administration (FDA) and the European Medicines Agency (EMA). Zolgensma^®^ and Luxturna^®^ are approved by the FDA, EMA, and the Pharmaceuticals and Medical Devices Agency (PMDA) in Japan, while Upstaza™ is approved by the EMA [2,3,4,5].

AAV vectors are distinguished by their ability to transduce both dividing and nondividing cells, their nonpathogenic nature, and the availability of tissue-specific serotypes. Despite these advantageous characteristics, several aspects of rAAV vectors warrant further investigation to enhance their therapeutic applications. These include their limited transgene cloning capacity, the requirement for high vector doses in therapy (typically ranging from 10^11^ to 10^14^ vg/kg), low cell transduction efficiency, immunogenicity, and the challenges associated with the complexity, difficulty, and high cost of vector production in vitro [6,7,8,9].

Low cell transduction efficiency is often compensated for by administering high doses of rAAV vectors. However, this approach is significantly constrained by issues related to immunogenicity and toxicity [10]. AAV vectors can provoke a systemic inflammatory response mediated by both the innate and adaptive immune systems. This response results in the secretion of pro-inflammatory cytokines and interferons, which drive the generation of antibodies and T cells specific to the AAV vectors. Activation of these immune mechanisms leads to the neutralization of the vectors and the elimination of AAV-transduced cells [11,12]. Additionally, due to the host’s immune response to the initial therapy and pre-existing immunity—where the prevalence of antibodies against AAV in humans ranges from 40% to 70% [13]—repeated dosing via systemic delivery remains unfeasible [14]. To address these challenges, research efforts are concentrated on improving cell transduction efficiency. Enhancing transduction efficiency can reduce the reliance on high vector doses, thereby mitigating the associated immunogenicity and toxicity [15]. This advancement is crucial for optimizing the therapeutic efficacy and safety of rAAV-based gene therapies.

The optimization of rAAV vectorology focuses on improving vector derivation methods, cloning of next-generation vectors characterized by reduced immunogenicity (e.g., capsid-modified vectors like tyrosine residue mutants [15]), efficient transduction (e.g., genome-modified vectors by 5′ end D-sequence deletion [15]), adjustable transgene expression (e.g., riboswitches [16]), and insertion of adjustable promoters (e.g., Tet-On reverse tetracycline-controlled transactivator (rtTA)-based system [17]).

Moreover, achieving the effective and safe use of rAAVs in gene therapy necessitates in-depth knowledge of the pharmacokinetics (PKs) and pharmacodynamics (PDs) associated with these vectors, particularly focusing on traditional drug and gene therapy medicinal product interactions [18]. Significant attention has been devoted to detailing the AAV life cycle and elucidating the mechanisms of transduction and transgene expression. This involves identifying host restriction factors that can affect the efficiency of rAAV transduction [19]. A genome-wide RNAi screening conducted by Mano et al. identified numerous genes that impact rAAV vector transduction efficiency [20]. Key host restriction factors involved in rAAV transmission include SETD8, CASP8A2, SOX15, TROAP, NPAT, PHC3, CHAF1A, SF3B, RTBDN, and WWC2 [20]. As demonstrated in our recent work, miRNAs are also significant factors influencing transduction efficiency [21]. Enhancing transduction efficiency, often through chemical agents and physical treatments, is a common approach. In our studies, we have shown that hyperthermia increases the transduction efficiency of rAAVs in ovarian cancer [22], colorectal cancer [23], and melanoma cells [24].

The use of drugs to improve rAAV transduction efficiency is also an interesting approach, with studies reporting the efficacy of proteasome inhibitors [25], sumo protein modifiers [26], and immunosuppressive agents [27]. In our recent study, we highlighted that various pharmacological agents have been used to improve AAV transduction at different stages of infection, suggesting potential interactions between pharmacotherapy and gene therapy agents. We underscore the necessity to evaluate both the pharmacokinetics and pharmacodynamics of rAAV [18]. However, the standard PK/PD framework may not be directly applicable to gene therapy products, making it necessary to evaluate the impact of drugs, particularly in the context of prevalent chronic diseases and polypharmacy, on the efficacy of these treatments.

It is well known that a substantial portion of the population regularly uses antifever and anti-inflammatory drugs, as well as systematic anticholesterol drugs. According to Business Research Insights, by 2028, the acetaminophen global market size will be USD 121.7 million [28], while the budesonide global market size will be USD 10.25 trillion [29], likely due to the large availability of OTC acetaminophen and the increased incidence of lung disease. In contrast, the increase in the population suffering from obesity and lipid disorders is a factor generating an estimated USD 22 trillion global market size for statins in 2032 [30]. Acetaminophen is used temporarily in cases of fever and pain of various origins [31]. Budesonide is indicated for specific conditions such as allergic rhinitis, asthma, and Crohn’s disease [32]. Simvastatin is commonly prescribed for familial hypercholesterolemia, hypertriglyceridemia, and a reduction in adverse cardiovascular events [30].

The primary objective of the present study is to evaluate the efficiency of rAAV transduction in the presence of commonly used medications. In the context of the increasing availability of gene therapy products based on rAAV vectors, a significant aspect of this research is to assess whether drugs such as acetaminophen, budesonide, and simvastatin increase rAAV transduction efficiency.

Future research will focus on several key areas to advance the field of clinical gene therapy. A novel research pathway is currently under development to dynamically support the progression of clinical protocols by integrating a critical evaluation of recombinant adeno-associated virus (rAAV) pharmacokinetics (PKs) and pharmacodynamics (PDs) with the role of pharmaceuticals as modulators of the transduction pathway. By integrating gene therapy protocols with existing pharmacological treatments, future studies aim to enhance clinical outcomes and explore novel methods to improve the effectiveness of current therapies. This comprehensive strategy will ultimately contribute to more effective and personalized treatment options.

## 2. Results and Discussion

**Figure 1:**
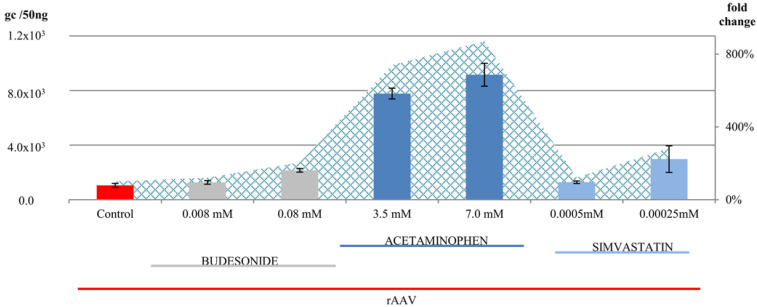
AAV/DJ transduction efficiency of the HEK-293 cell line in the presence of budesonide (gray bars), acetaminophen (dark blue bars), and simvastatin (light blue bars). Results are presented as the genome copy number ± standard deviation. The control group (red bar) was the HEK-293 cells transduced with rAAV/DJ alone. The area plot shows the fold change in transduction efficiency of HEK-293 cells with the rAAV/DJ vector in the presence of drugs compared with the control and is expressed as a percentage.

**Figure 2:**
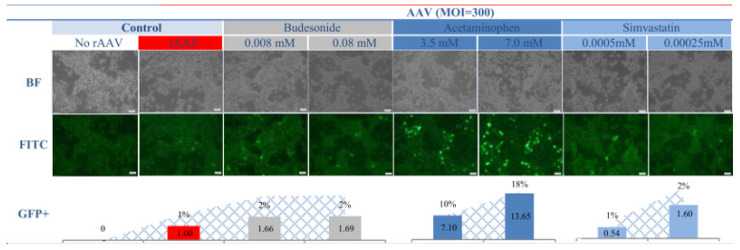
Transduction of HEK-293 cell line with rAAV/DJ at an extremely low MOI (300) in the presence of budesonide (gray color), acetaminophen (dark blue color), and simvastatin (light blue color). BF—bright field, FITC—fluorescent field, scale bar—50 µm. The control group (red color) includes samples transduced with rAAV/DJ without drugs. The column graph shows the number of GFP-positive (GFP+) cells as a fraction of the total number of cells present in the field of view. The area plot shows the fold change in the transduction efficiency of HEK-293 cells with the rAAV/DJ vector in the presence of drugs compared to the control and is expressed as a percentage.

**Figure 3:**
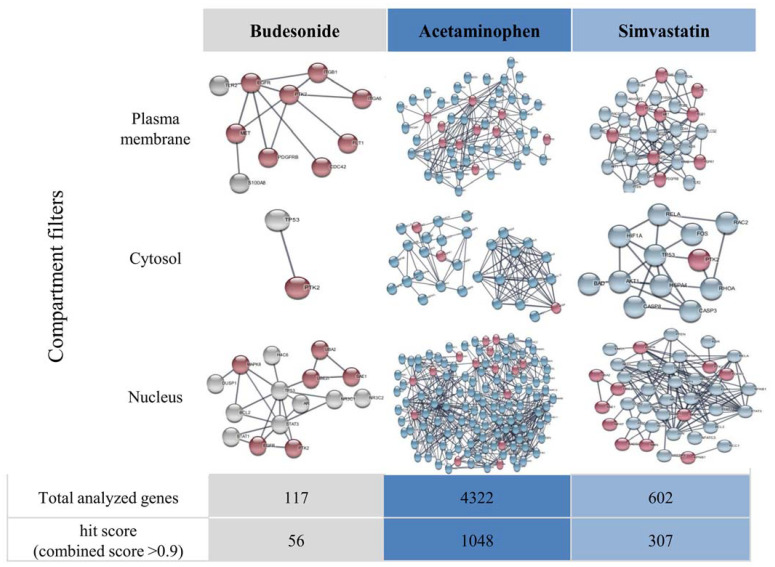
Correlation analysis between rAAV transmission genes (red nodes) and drug-modified genes (gray, dark blue, or light blue for budesonide, acetaminophen, or simvastatin, respectively) using a STRING database [4]. The obtained hits were divided into selected cellular compartments (plasma membrane, cytosol, and nucleus) presented horizontally in the graph. The hit score constitutes the combined score > 0.9, which is computed by combining the probabilities from the different evidence channels and correcting for the probability of randomly observing an interaction.

**Figure 4:**
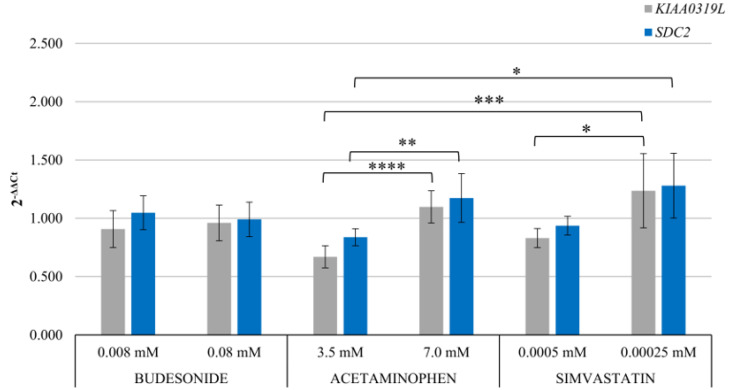
Expression of rAAV receptors, KIA319LL and SDC2, in HEK-293 cells after treatment with budesonide, acetaminophen, and simvastatin. The statistical significance was analyzed between the samples treated with medicines (* *p* < 0.05, ** *p* < 0.01, *** *p* < 0.0001, and **** *p* < 0.00001) using a Kruskal–Wallis test and a subsequently performed post hoc test, Dunn’s test.

Recombinant AAV vectors have become integral components of numerous experimental studies and clinical trials. Excellent review papers published in recent years have effectively outlined the biology and potential of rAAVs as carriers in gene therapy protocols [33]. At the same time, there is a growing body of work addressing the PKs/PDs of rAAVs, the necessity to develop vector biodistribution models, and the importance of potential interactions between medications already taken by patients and the implemented gene therapy medicinal products [18,34,35]. The topic of potentiating the efficiency of rAAV transfer into cells, improving expression activity, and extending the duration of expression remains relevant [36,37]. Improvement in transduction efficiency is achieved through the modulation of the transduction environment (hyperthermia or ultrasound) and the use of various traditional drugs (rapamycin or rituximab) [23,38,39,40]. Many pharmacological substances have been used to boost AAV transduction at various stages of infection, suggesting possible interactions between pharmacotherapy and gene therapy agents [41]. As these works illustrate, achieving targeted and efficient transduction is a complex process influenced by numerous physicochemical factors, which can be supplemented in experimental systems. It is also important to note that in gene therapy trials, the clinical condition of patients and their burden of diseases can also affect the efficiency of rAAV treatment. Experiences during the time of COVID-19 have shown that the efficiency of the transfer of vector vaccine doses was also affected by the patient’s health status and, very often, the presence of comorbidities requiring pharmacotherapy. Observations suggest a research hypothesis that patients treated with classical drugs may respond differently to gene therapy products in terms of efficiency and expression. This paper presents a study indicating that drugs commonly used in standard medical practice such as anti-inflammatory (budesonide), antipyretic (acetaminophen), and anticholesterol (simvastatin) drugs affect the transfer of rAAV into cells of the well-recognized HEK-293 cell line. To study the transduction efficiency, we used a quantitative method, namely real-time qPCR, and visualized rAAV-positive cells through fluorescence microscopy. Based on the available literature, our study is the first to report the effects of acetaminophen, budesonide, and simvastatin on rAAV transduction into human cells. As illustrated in Figure 1 and Figure 2, these drugs increase transduction efficiency at very low MOIs. Transduction efficiency, measured as the number of copies of ITR sequences present in rAAV, increased by 9-fold for acetaminophen (7.0 mM), 2-fold for budesonide (0.08 mM), and 3-fold for simvastatin (0.00025 mM) (Figure 1). It is important that the presence of rAAV in transduced HEK-293 cells is also accompanied by the expression of the GFP reporter gene. Figure 2 illustrates GFP-positive cells observed after rAAV transduction in the presence of drugs. The study used the rAAV/DJ-CMV-eGFP vector. The AAV/DJ vector was created by genetic engineering, recombining eight wild-type AAV serotypes. This vector exhibits a broader range of tropism compared to the original serotypes and demonstrates superior transduction efficiency [42,43]. Additionally, it has a lower level of immunogenicity compared to other rAAV vectors. In previous studies, we have shown that rAAV/DJ efficiently transduces ovarian cancer [22], colorectal cancer [23], and melanoma cells [24]. The high response of HEK-293 cells to the rAAV in the presence of drugs raises questions about potential molecular mechanisms underlying drug-dependent transduction. To explore this, we performed an association analysis of the functional interdependence of genes involved in rAAV transduction and their relationship with genes modulated by the studied drugs. The drugs used are among the classic ones used in therapy, and many papers have characterized the complex signal transduction pathways and specific signatures of drug-activated genes [44,45,46,47]. Rich sources of information are online repositories that allow for the exploration of the relationship between genes. In our study, we used DSigDB because it contains the largest number of drug- and compound-related gene sets. In our work, an informatics analysis was conducted to investigate the interactions between genes involved in rAAV transmission and the cellular response to the studied drugs. Current knowledge of rAAV transmission was considered, including the importance of receptor signaling (AAVR and KIAA0319L) as postulated by Pillay et al. [48]. The results are summarized in Figure 3 and Figure 4.

This study indicates that a significant amount of interaction between transmissibility genes (red nodes) and drug-modified genes is observed for genes belonging to the plasma membrane compartment (Figure 3). Of note is the pivotal role of EGFR (a gene related to rAAV transduction, marked in red) in its association with genes whose expression is modulated by all the studied drugs (budesonide, acetaminophen, and simvastatin). Epidermal growth factor receptor protein tyrosine kinase (EGFR-PTK) phosphorylates FK506 binding protein (FKBP52), inhibiting second-strand viral DNA 2 (AAV2) synthesis and transgene expression [49]. Attention should also be given to *S100A8*, a gene frequently modified by anti-inflammatory drugs such as budesonide and simvastatin. This gene shows an interaction with the gene encoding the receptor for AAV, HGFR (MET). The least number of interactions between genes of both groups was exhibited by budesonide, while acetaminophen demonstrated the highest number of interactions. This variation may be attributed to the number of genes available in the selected analysis. As depicted in Figure 3, acetaminophen is characterized by multidirectional interactions in the cell at the level of gene expression. A comprehensive understanding of the roles of these analyzed genes in the transmission of rAAV in the presence of drugs requires further studies involving different cell lines, animal models, and advanced research methodologies. In the research panel assessing the influence of pharmacotherapeutics on the expression of reference receptors for rAAV (KIAA0307L and SDC2), drug-induced induction of expression was observed, notably with acetaminophen and simvastatin (Figure 4). It was shown that there was an increase in the expression of both receptors, and this effect was dependent on the dosage of the administered drug.

## 3. Conclusions

Our work, for the first time, indicates that improvements in the transduction efficiency of well-recognized rAAV vectors in medicine can be achieved through the use of anti-inflammatory, antipyretic, and anticholesterol drugs. The capacity of these drugs to modulate the advancement of rAAV gene therapy protocols can significantly expand the applications of rAAV in human medicine. This article underscores the importance of conducting medication reviews for patients eligible for gene therapy medicinal products, highlighting the potential impact of pharmacotherapeutics (including those available over the counter) on the efficiency of rAAV vector transduction. This approach promises enhanced safety, potentially lower dosages, and improved transduction and gene expression.

## 4. Materials and Methods

### 4.1. Reagents and Materials

Dulbecco’s Modified Eagle Medium (DMEM), fetal bovine serum (FBS), and an antibiotic antimycotic solution (AAS) were procured from Gibco (Gibco, Paisley, UK). The ITR primers and probe were sourced from IBB Poland. The recombinant AAV vector rAAV/DJ-CMV-eGFP (cat. no: 7101) was purchased from Vector Biolabs (Vector Biolabs, Malvern, PA, USA). The AAV-293 (HEK-293) cell line (cat. No: 240073) was obtained from Agilent (Agilent Technologies Inc., Santa Clara, CA, USA). The TaqMan™ Universal Master Mix II was acquired from Thermo Fisher Scientific (Applied Biosystems^TM^, Waltham, MA, USA). All medications were obtained from the pharmacy, including a 10 mg/mL acetaminophen solution for infusion (Paracetamol, B. Braun), 0.5 mg/mL budesonide nebulizer suspension (Nebbud, TEVA Pharmaceuticals), and 10 mg simvastatin tablets (Simvasterol, Polpharma).

#### Preparation of Drug Solutions

The preparation of drug solutions involved the following procedures: The 10 mg/mL acetaminophen solution for infusion (Paracetamol, B. Braun) and the 0.5 mg/mL budesonide nebulizer suspension (Nebbud, TEVA Pharmaceuticals) were both used directly from their original packaging. Both of these products are sterile and free from mechanical contaminants, making them suitable for use in cell cultures.

For the simvastatin solution, two 10 mg Simvasterol tablets were crushed in a mortar. The micronized powder was then mixed with 10 mL of 70% ethanol and shaken for 20 min. The solution was subsequently filtered through a qualitative filter. Subsequently, it was subjected to sterile filtration (0.22 µm) in a chamber with a laminar flow of sterile air. A solution with a concentration of 2 mg/mL (4800 µM) was obtained.

### 4.2. rAAV Transduction

The HEK-293 cells (2.5 × 10^5^) were seeded in 4.0 mL of medium (DMEM, 10% FBS, 1% AAS) in a 6 cm diameter plate (NUNC) and cultured at 37 °C in a 5% CO_2_ atmosphere for 72 h. For transduction, the medium was replaced with DMEM containing 2% FBS. HEK-293 cells were then treated with drug solutions of budesonide, acetaminophen, or simvastatin in quantities ensuring the following drug concentrations: 0.08 and 0.008 mM for budesonide, 3.5 and 7.0 mM for acetaminophen, and 0.0005 and 0.00025 mM for simvastatin. Subsequently, rAAV vectors were introduced into the culture medium at a multiplicity of infection (MOI) of 300 genome copies. The cells were then moved to a 37 °C environment with a humidified 5% CO_2_ atmosphere for 96 h. Finally, the cells were harvested and HEK-293 cells were transduced with rAAV/DJ-CMV-eGFP, with no drugs used as a control.

### 4.3. Measurement of Transduction Efficiency

A. Real-time PCR for examination of rAAV genome copy number

Total DNA was isolated using the High Pure Viral Nucleic Acid Kit (Roche Diagnostics GmbH, Mannheim, Germany). To determine the rAAV genome copy number, TaqMan assays were designed using a probe and primers for the ITR region [50]. The analysis used the TaqMan 5′-CACTCCCTCTCTGCGCGCTCG-3′ probe featuring 6-FAM, TAMRA, and the following primers: forward 5′-GGAACCCCTAGTGATGGAGTT-3′ and reverse 5′-CGGCCTCAGTGAGCGA-3′. A standard curve was created using serial dilutions of plasmid DNA pAAV-hrGFP (Part No. 240074; Agilent Technologies, Santa Clara, CA, USA). On the basis of the obtained course of the standard curve, the efficiency of the reaction was determined, which was in the range of 95–105%. Each qPCR reaction had a total volume of 10 µL and contained 50 ng of DNA. The reaction ran under the following conditions: 50 °C for 2 min, and 95 °C for 10 min, followed by 40 cycles of 95 °C for 15 s, and 60 °C for 60 s. The StepOnePlus™ Real-Time PCR System (Applied Biosystems, Thermo Fisher Scientific) was used to perform real-time PCR.

B. Fluorescent imaging

Documentation of the GFP-positive cells of the HEK-293 cell lines was made using an inverted optical microscope (IX53, Olympus, Tokyo, Japan) with the pE-300white (CoolLED) illumination system. Observations were made in both bright field and with the use of a fluorescent FITC filter (fluorescein isothiocyanate) at ×10 magnification. Imaging and analysis were performed using cellSens Dimension 1.18 software (Olympus). All measurements were conducted 96 h after transduction, before cell harvesting.

### 4.4. Assessment of the Expression of rAAV Receptors

The HEK-293 cells (1.0 × 10^6^) were seeded in 4.0 mL of medium (DMEM, 10% FBS, 1% AAS) in a 6 cm diameter plate (NUNC) and cultured at 37 °C in a 5% CO_2_ atmosphere for 24 h. Following this incubation period, the medium was replaced with DMEM containing 2% FBS. Subsequently, the HEK-293 cells were treated with drug solutions of budesonide, acetaminophen, or simvastatin, with concentrations set at 0.08 and 0.008 mm for budesonide, 3.5 and 7.0 mm for acetaminophen, and 0.0005 and 0.00025 mm for simvastatin. The cells were then maintained at 37 °C in a humidified 5% CO_2_ atmosphere for 4 h. Following drug exposure, the medium was replaced with DMEM containing 2% FBS without drug solutions. Finally, after an additional 24 h of incubation, the cells were harvested. Total RNA was isolated using the PureLink™ RNA Mini Kit (Invitrogen^®^, Waltham, MA, USA). The conversion of RNA into cDNA was assessed using the High Capacity RNA-to-cDNA kit (Thermo Fisher Scientific). Taqman assays from Thermo Fisher Scientific (AAVR: Hs00967343_m1, HSPG1: Hs01081432_m1) were utilized, with ACTB (Hs01060665_g1) serving as the internal reference. Quantitative PCR (qpcr) determined expression levels relative to ACTB. Control samples consisted of cells untreated with drugs. The calculations were performed for the tested and calibration samples using the ΔΔCt method.

## 5. Visualization of Functional Gene Interactions in Drug-Exposed Cells

The STRING database was used for the construction of protein interaction diagrams for the examined transcripts [51]. The relationship between rAAV transmissibility genes and drug-modified genes was investigated. The following genes were selected as related to rAAV vectors transduction: *KIAA0319L*, *PTK2*, *SDC2*, *HSPG2*, *FGFR1*, *STX5*, *EGFR*, *MET*, *CD9*, *PDGFRB*, *RPSA*, *TLR9*, *FLT1*, *ITGA5*, *ITGB1*, *CDC42*, *ARF1*, *TROAP*, *VPS29*, *VPS52*, *VPS54*, *ATP2C1*, *WASHC4*, *GPR108*, *UBA2*, *UBE2I*, *SAE1*, *KPNB1*, *CASP8AP2*, *SOX15*, *U2SURP*, *NPTN*, *PHF5A*, *KMT5A*, *PHC3*, *SF3B1*, *U2AF1*, *FKBP4*, *NPAT*, *NBN*, *RAD50*, *MRE11*, and *MAPK8* (marked in red). The list of genes whose expression is regulated by drug administration (budesonide 117, acetaminophen 4322, and simvastatin 602) was obtained from the DSigDB database [44]. The analysis was performed using a minimum required interaction score > 0.900, and genes functionally representative of cellular compartments (plasma membrane, cytosol, and nucleus) were selected from the obtained hits.

## 6. Statistical Analysis

The statistical analysis was conducted using GraphPad Prism 9 (GraphPad Software, La Jolla, CA, USA). The significance of differences between the mean values of compared samples was calculated utilizing a Kruskal–Wallis test (α = 0.05). A post hoc test, Dunn’s test (α = 0.05), was employed to assess the statistical significance of the differences. Experiments were performed in triplicate. The level of significance was indicated as * *p <* 0.05; ** *p <* 0.01; *** *p <* 0.001; and **** *p <* 0.0001.

## Data Availability

Data will be made available on request.
